# Novel HER3/MUC4 oncogenic signaling aggravates the tumorigenic phenotypes of pancreatic cancer cells

**DOI:** 10.18632/oncotarget.3912

**Published:** 2015-05-16

**Authors:** Imayavaramban Lakshmanan, Parthasarathy Seshacharyulu, Dhanya Haridas, Satyanarayana Rachagani, Suprit Gupta, Suhasini Joshi, Chittibabu Guda, Ying Yan, Maneesh Jain, Apar K. Ganti, Moorthy P. Ponnusamy, Surinder K. Batra

**Affiliations:** ^1^ Department of Biochemistry and Molecular Biology, University of Nebraska Medical Center, Omaha, NE, USA; ^2^ Department of Genetics, Cell Biology and Anatomy, University of Nebraska Medical Center, Omaha, NE, USA; ^3^ Eppley Institute for Research in Cancer and Allied Diseases, University of Nebraska Medical Center, Omaha, NE, USA; ^4^ Department of Medicine, VA Nebraska Western Iowa Health Care System, Omaha, NE, USA; ^5^ Department of Internal Medicine, University of Nebraska Medical Center, Omaha, NE, USA; ^6^ Eppley Institute for Research in Cancer and Allied Diseases, Fred & Pamela Buffett Cancer Center, University of Nebraska Medical Center, Omaha, NE, USA

**Keywords:** pancreatic cancer, HER2, HER3, MUC4, KPC model

## Abstract

Several studies have demonstrated that MUC4 is involved in progression and metastasis of pancreatic cancer (PC). Here, we report that HER3/MUC4 interaction in HER2 low cells is critical in driving pancreatic tumorigenesis. Upon HER2 knockdown, we observed elevated expression of HER3 and MUC4 and their interactions, which was confirmed by immunoprecipitation and bioinformatics analyses. In paired human PC tissues, higher percentage of HER3 positivity (10/33, 30.3%; *p* = 0.001) was observed than HER2 (5/33, 15.1%; *p* = 0.031), which was further confirmed in spontaneous mice (KPC; Kras^G12D^; Trp53^R172H/+^; Pdx-Cre) tumors of different weeks. Mechanistically, increased phosphorylation of ERK and expression of PI3K and c-Myc were observed in HER2 knockdown cells, suggesting a positive role for HER3/MUC4 in HER2 low cells. Further, HER2 knockdown resulted in increased proliferation, motility and tumorigenicity of PC cells. Consistently, transient knockdown of HER3 by siRNA in HER2 knockdown cells led to decreased proliferation. These observations led us to conclude that HER3 interacts with MUC4 to promote proliferation in HER2 low PC cells. Further, deficiency of both HER2 and HER3 leads to decreased proliferation of PC cells. Hence targeting these newly identified HER3/MUC4 signals would improve the PC patients survival by intercepting MUC4 mediated oncogenic signaling.

## INTRODUCTION

Epidermal growth factor receptors (EGFR) are a family of cell surface proteins, which consists of human EGFR (HER1), ERBB2 (HER2), ERBB3 (HER3) and ERBB4 (HER4) [1–4]. These molecules contribute towards the aggressiveness of several cancers, including pancreatic cancer [5–8]. EGF receptors have an extracellular domain that binds to ligands, a α-helical transmembrane region, and a cytoplasmic protein tyrosine kinase domain [9]. EGF receptors bind to specific ligands such as epidermal growth factor (EGF), transforming growth factor α (TGF-α), amphiregulin, epiregulin, betacellulin, heparin-binding EGF and epigen which induce conformational changes, and convert inactive monomers to active homodimers/heterodimers [1]. Autophosphorylation of tyrosines within the regulatory tail at the c-termini of the receptors is required for downstream signaling [2, 10]. HER2 amplification and overexpression are often detected in cancer and have been shown to contribute to tumor development [11]. HER2/HER3 hetero-dimerization has also been shown to be important for cancer development [12, 13]. HER3 does not have the intrinsic tyrosine kinase activity and hence needs to dimerize with other HER family members to induce downstream signaling [14]. In addition to HER3/EGFR dimerization, HER3/HER2 dimerization is also observed in many tumors, it suggesting that HER3 also is required for cancer cell survival and proliferation [13, 15]. Several recent studies have indicated the importance of HER3 in various cancers including pancreatic cancer [1, 10, 16–18]. For instance, HER3 induces strong downstream oncogenic signaling by activating PI3K in many tumors and escape various EGF receptors targeted therapies. Hence emerging attentions to develop specific anticancer agent against HER3 to inhibit the HER3 oncogenic signaling [19, 20]. Grb2 is an adaptor protein, which has been shown to be associated with HER family proteins [21]. The signaling adaptor protein of Grb2 contains SH domain, which is required for interactions of various phosphorylated proteins such as HER family proteins [22].

Several studies have shown that EGF receptor interacts not only with their own family members, but also with other proteins [4, 23]. For example, we reported that MUC4 mucin interacts with HER2 and stabilizes it, induce proliferation and metastasis of pancreatic [24] and ovarian cancer cells [25]. MUC4 is a type I transmembrane glycoprotein, and its expression has been shown to strongly correlate with the aggressiveness of various cancers including pancreatic cancer [26, 27]. MUC4 is differentially expressed at various stages of pancreatic cancer [5, 28]. We have previously reported that MUC4/HER2 interaction plays an important role for proliferation and metastasis of pancreatic cancer cells [24]. However, few questions that remained unanswered include i) what is the interplay between MUC4 and HER family proteins, and ii) what are the contributions of other HER family members to the proliferation of pancreatic cancer cells.

In the present study, we aimed to analyze the importance of HER3 in the HER2 low pancreatic cancer. Our observation showed that HER3 is relatively overexpressed in pancreatic cancer patient tissues compared to HER2. Upon HER2 knockdown, HER3 and MUC4 appear to be overexpressed leading to increased proliferation and tumorigenesis of pancreatic cancer cells. Thus, our data suggest that HER3 interacts with MUC4 and is involved in the proliferation of pancreatic cancer cells in the absence of HER2. Consequently, MUC4 could drive cancer progression by engaging HER3 oncogenic signaling in HER2 low pancreatic cancer cells.

## RESULTS

### Loss of HER2 upregulates HER3 and MUC4 in pancreatic cancer cells

**Figure 1:**
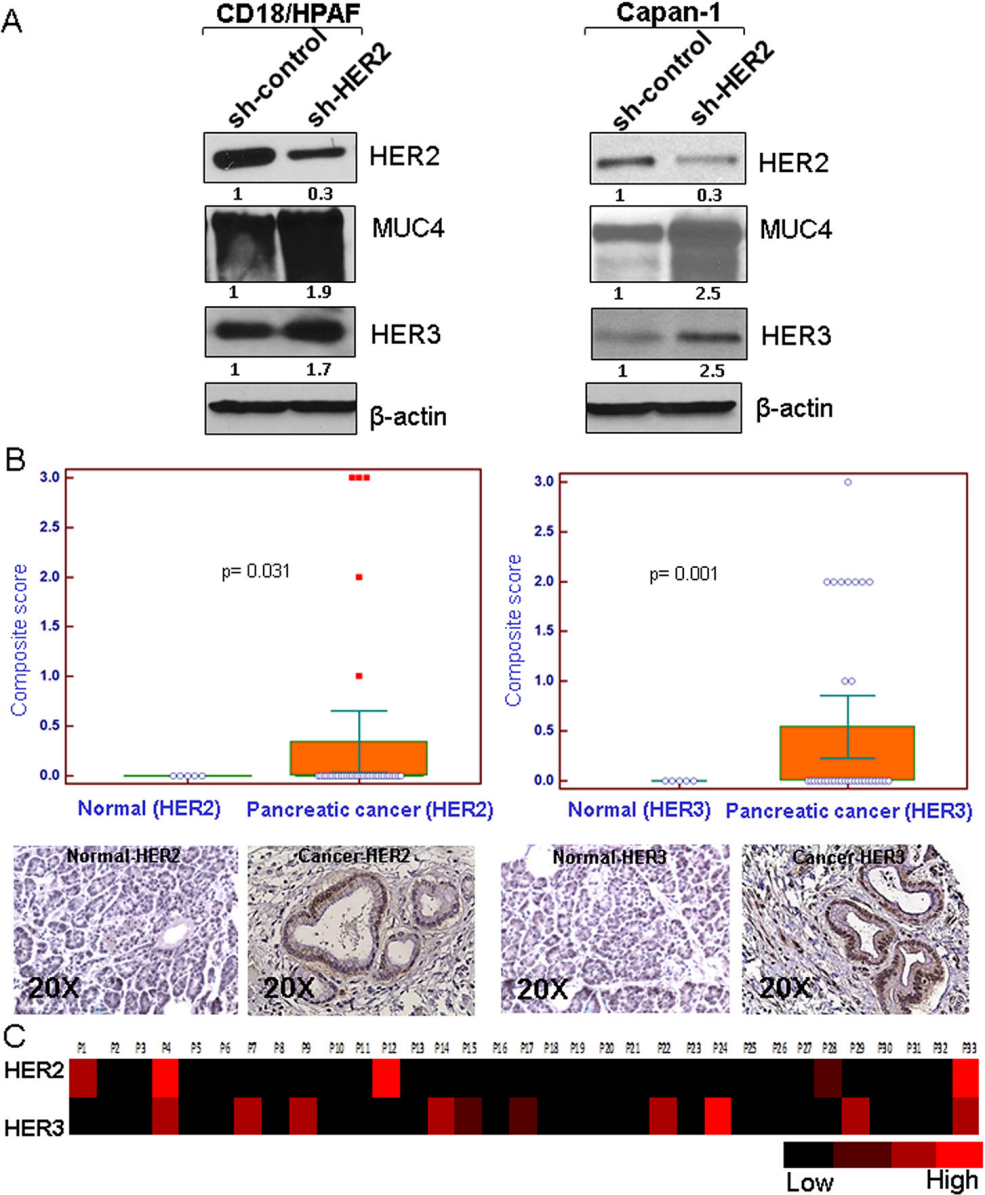
Effect of HER2 knockdown on HER3 and MUC4 expression and relative expression of HER2 and HER3 in pancreatic cancer patient tissues **A.** HER2 stable knockdown (~70%) in two pancreatic cancer cells (CD18/HPAF and Capan-1) leads to increased expression of HER3 and MUC4 in comparison to control shRNA treated cells as demonstrated by western blot analysis. β-actin was used as loading control. **B.** Immunohistochemistry was performed in pancreatic cancer patient tissues obtained from Rapid Autopsy program at UNMC. The results indicate higher incidence of HER3 (10/33, 30.3%; *p* = 0.001) overexpression than HER2 (5/33, 15.1%; *p* = 0.03) in pancreatic cancer (figure magnification 20X). **C.** Heat map of composite scores indicate that HER3 expression is relatively more than HER2.

We and others have shown that HER2 interacts with MUC4 in pancreatic and ovarian cancer cells, and MUC4 knockdown results in decreased HER2 signaling and cell proliferation [23–25]. To elucidate the HER2 independent mechanism in the regulation of pancreatic cancer cell proliferation, two aggressive pancreatic cancer cell lines (CD18/HPAF and Capan-1) were chosen, which express both HER2 and MUC4 endogenously. These cell lines were stably transfected with HER2 shRNA (three different sequence targets), resulted in decreased HER2 expression than control shRNA cells as demonstrated by western blot analysis (Figure 1A and Supplementary Figure 1A). Following HER2 knockdown, the expression profile of HER family members namely EGFR, HER3 and HER4 was examined. Interestingly, we observed that in the HER2 knockdown cells, HER3 and MUC4 were upregulated in comparison to the scrambled control cells, as determined by western blot analyses as shown in Figure 1A and Supplementary Figure 1A. We also observed an increase of Grb2 expression in HER2 knockdown cells relative to scrambled control cells (Supplementary Figure 1B). In contrast, there were no changes in the expression of EGFR and HER4 in both HER2 knockdown pancreatic cancer cells (Supplementary Figure 1B). Previous reports from our lab have shown that MUC4 is required for progression, and metastasis of pancreatic cancer [27, 29]. In this study, we have demonstrated increased expression of HER3 in HER2 knockdown cells, which may associate with MUC4 for pancreatic cancer cell proliferation.

### Relative expression of HER2 and HER3 in pancreatic cancer patient tissues

HER2 and HER3 heterodimerization is most effective among other EGFR family members in terms of strength of interaction, tyrosine phosphorylation and their downstream oncogenic signal in variety of cancer [12, 30]. In order to determine the relative expression and clinical relevance of HER2 and HER3 in pancreatic cancer, we utilized the pancreatic cancer patients tumor tissues (obtained from Rapid Autopsy program at UNMC) for immunohistochemical analysis. The incidence of HER3 expression was higher (10/33, 30.3%; *p* = 0.001) as compared to that of HER2 expression (5/33, 15.1%; *p* = 0.031) (Figure 1B). Further, the relative expression between HER2 and HER3 positive pancreatic tumor was analyzed, and the results show that HER3 expression was relatively higher than HER2 (Figure 1B). To obtain a comparative pictorial representation of the relative expression between HER2 and HER3, heat map analysis was performed (Figure 1C). In support of this study, in pancreatic cancer HER3 is overexpressed to a greater degree (fold change 5.14) than HER2 (fold change 3.05) as indicated in the Oncomine database.

### Co-localization of MUC4/HER3 in pancreatic cancer cells and KPC tumor tissues (KPC; Kras^G12D^; Trp53^R172H/+^; Pdx-Cre) and interaction of MUC4 and HER3 in pancreatic cancer cells

**Figure 2:**
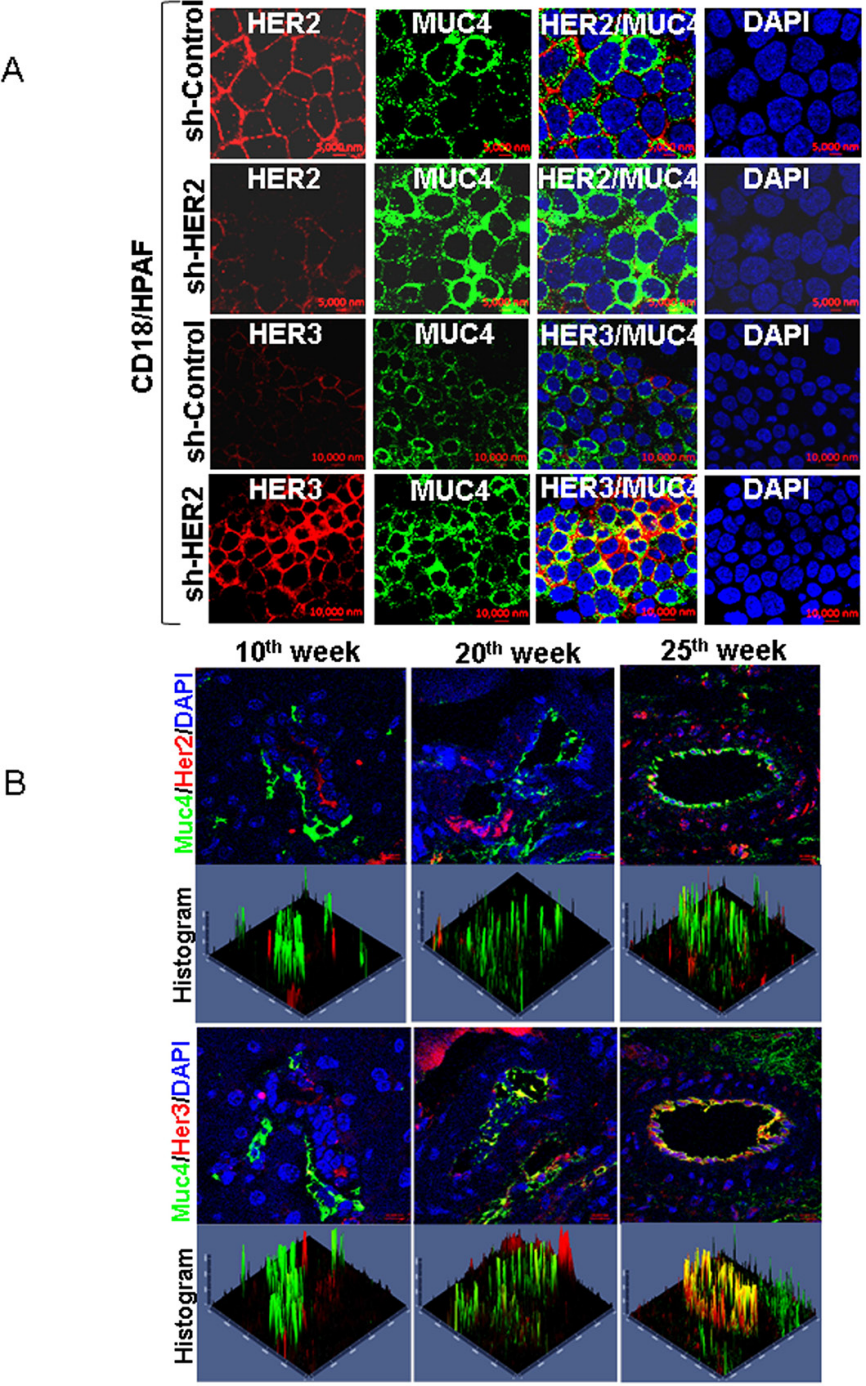
Co-localization of MUC4 and HER3 in pancreatic cancer cells and KPC tumor tissues **A.** Confocal analysis show that MUC4 is strongly co-localized with HER3 in HER2 knockdown CD18/HPAF cells. Further expression of HER2 in HER2 silenced cells and elevated expression of HER3 and MUC4 was observed in CD18/HPAF cells. **B.** Similarly, Muc4/Her3 co localization was observed in tumor tissues of Kras and p53 (Kras^G12D^; Trp53R172H^−/+^; Pdx-1-Cre) mediated pancreatic cancer progression mice model. This results show that co-expression of Muc4/Her3 is relatively higher than Muc4/Her2 in pancreatic cancer progression mice model (10^th^ week, 20^th^ week and 25^th^ week).

In order to find out the distribution of MUC4 and HER3 in pancreatic cancer cells, we performed confocal microscopy analysis. The results show that MUC4 is strongly co-localized with HER3 in HER2 knockdown CD18/HPAF cells (Figure 2A). Similarly decreased expression of HER2 was observed in HER2 knockdown cells than scrambled control CD18/HPAF cells (Figure 2A). We have also investigated the significance of Muc4, Her2 and Her3 during triple transgenic mouse pancreatic cancer progression model (KPC; Kras^G12D^, Trp53^R172H−/+^; and Pdx-Cre). Interestingly, we observed increased co-localization of Muc4/Her3 in various stages (10^th^, 20^th^ and 25^th^ weeks) of pancreatic cancer progression in mice tumor tissues than Muc4/Her2 expression (Figure 2B). These results suggest a potential involvement of MUC4/HER3 interaction in pancreatic cancer progression.

**Figure 3:**
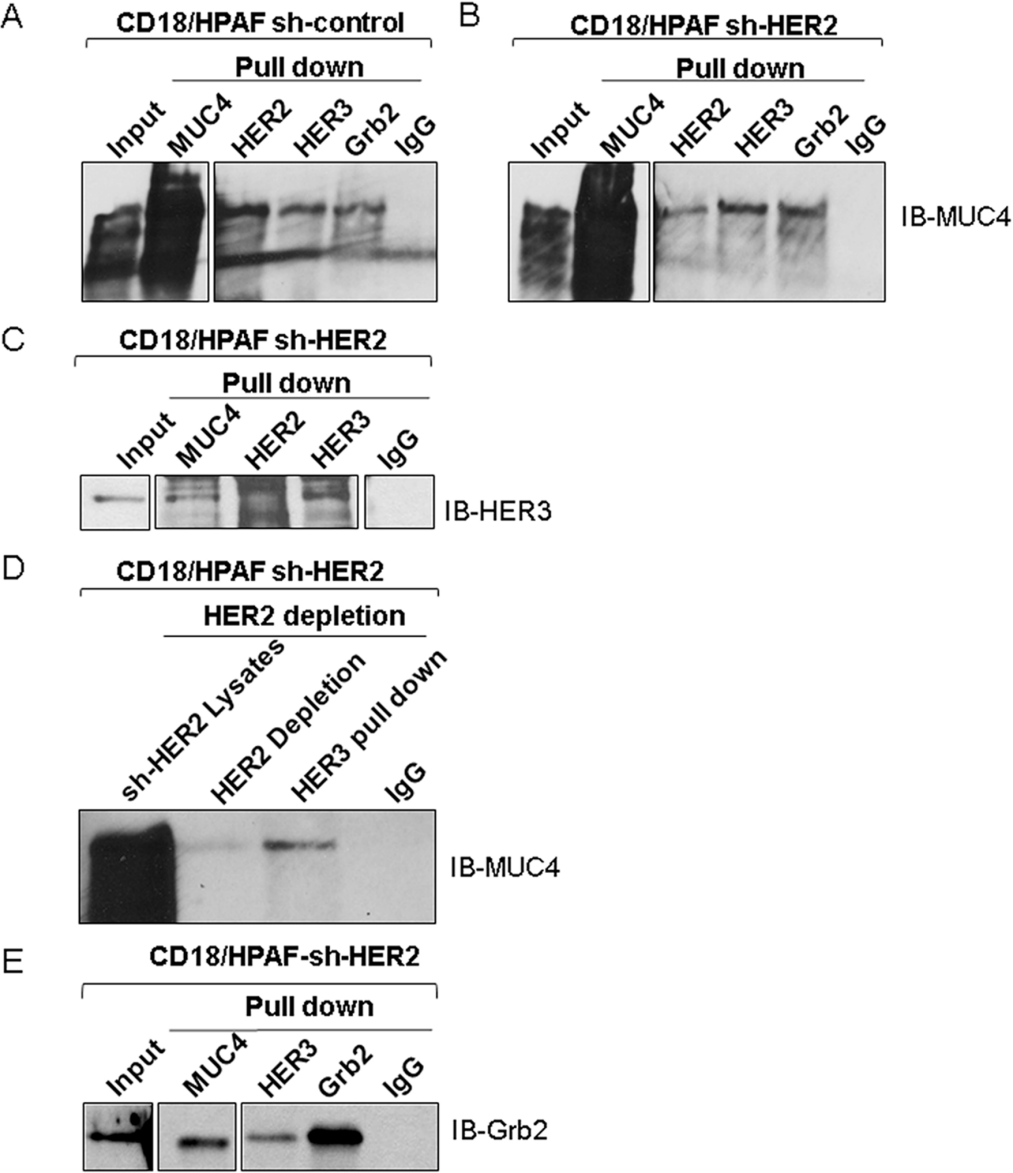
Association of MUC4, HER2 and HER3 in pancreatic cancer cells **A and B.** The co-immunoprecipitation results represent that MUC4 interacts with HER3 and Grb2 in HER2 knockdown and scrambled control of CD18/HPAF cells. **C.** Reciprocal immunoprecipitation results show that HER3 strongly interacts with MUC4 in HER2 knockdown cells. **D.** Represents that MUC4 and HER3 interaction in HER2 negative background by HER2 immuno depletion method. **E.** Represents that anti-MUC4 and anti-HER3 antibpodies pull down Grb2 in HER2 knockdown CD18/HPAF cells.

HER2 heterodimerizes with EGFR, HER3, and HER4, as well as with other proteins like MUC4 which contain EGF-like domains [31]. Since, MUC4 acts as an oncogene during the progression and metastasis of pancreatic cancer [28], we hypothesized that in the absence of HER2, HER3 may interact with MUC4 to promote pancreatic cancer cell proliferation. To test this hypothesis, we analyzed the MUC4/HER3 interaction. Reciprocal co-immunoprecipitation assay showed that HER3 interacts with MUC4 in sh-Control (Figure 3A) and HER2-knockdown pancreatic cancer cells (Figure 3B and 3C). In order to analyze the MUC4/HER3 interaction in a HER2 negative background, we further eliminated residual HER2 from the CD18/HPAF sh-HER2 cell lysate using immunodepletion method (precipitated HER2). HER3 was then immunoprecipitated from the HER2 depleted samples and probed for MUC4 (Figure 3D). As shown in Figure 3D, MUC4 was detected in the HER3 immunoprecipitates (HER2 depleted), indicating an interaction between HER3 and MUC4. These results suggest that HER3 is overexpressed and associates with MUC4 in a HER2 independent manner. In addition, elevated Grb2 expression (Supplementary Figure 1B), and interaction with HER3 and MUC4 was observed upon loss of HER2 (Figure 3B and 3E). These observations suggest that HER3/MUC4 interaction may recruit adaptor molecule Grb2, thereby potentially inducing downstream signaling, leading to increased pancreatic cancer cell proliferation.

### Bioinformatic studies for MUC4 and HER3 interaction

To infer potential interactions between the domains of MUC4 and HER3 proteins, we used a bioinformatic method that could predict the likelihood with which any two protein domains could interact. We found that the Epidermal Growth Factor-like (EGF-2) domain of MUC4 can interacts with HER3 with a confidence score of 70% [32]. In addition, the sequence identity between HER2 and HER3 is 47% (Supplementary Figure 2A) and these two proteins share the same domain structure (Supplementary Figure 2B), except that HER2 active site residue (D845) is missing in HER3 [33].

### Hyperproliferation of HER2 knockdown cells and downstream signaling

**Figure 4:**
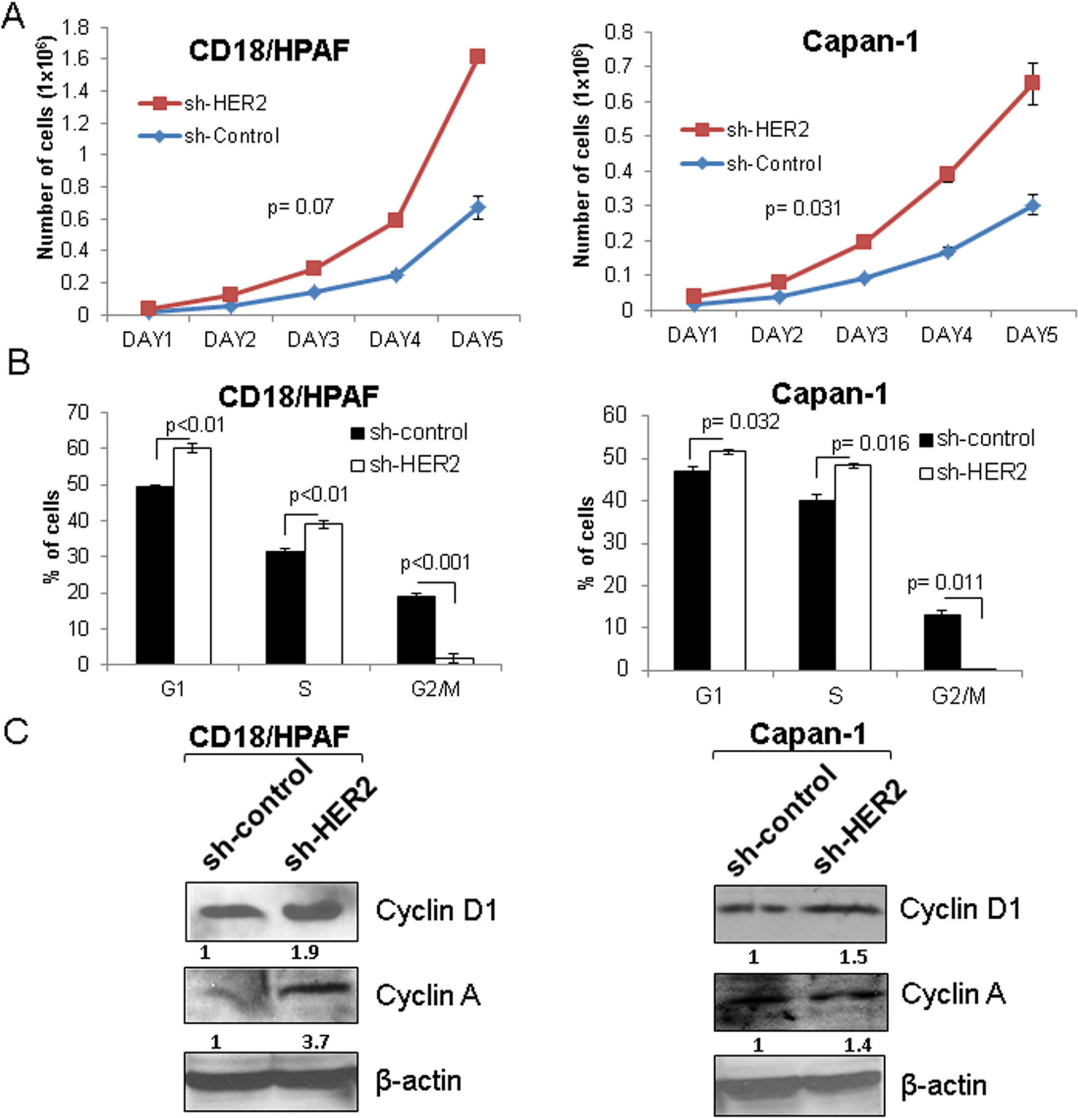
Effect of HER2 silencing on the proliferation of pancreatic cancer cells **A.** HER2 knockdown leads to increased proliferation in two stable knockdown pancreatic cancer cell lines (CD18/HPAF and Capan-1) as demonstrated by growth kinetics assay. **B.** Similarly, FACS analysis also indicates the increased S-phase cells in HER2 knockdown than scrambled control cells. **C.** Western blot results show that Cyclin D1 and Cyclin A are increased in HER2 knockdown in CD18/HPAF and Capan-1 cells.

Though HER2 plays an oncogenic role in many cancers [9, 25, 34], we observed an increased rate of proliferation in the HER2 knockdown cells as compared to scrambled control CD18/HPAF and Capan-1 cells by performing growth kinetics assay (Figure 4A and Supplementary Figure 3). Similarly, cell cycle studies show that HER2-knockdown cells have a higher percentage of cells in the S-phase (48 h) (Figure 4B). These results indicate that the HER2-knockdown cells exhibit increased proliferative ability compared to scrambled control cells. Furthermore, HER2 knockdown cells had a more rapid G2/M transition than scrambled control cells (*p < 0.001)* (CD18/HPAF) and *p = 0.011*(Capan-1)), which also indicates that HER2 knockdown cells have higher mitotic activity as compared to scrambled control cells (Figure 4B). Moreover, western blot results showed an increased level of cell cycle proteins Cyclin D1 and Cyclin A in HER2-knockdown cells compared to the scrambled control cells, indicating their involvement in the elevated proliferative activity of HER2-knockdown cells (Figure 4C).

**Figure 5:**
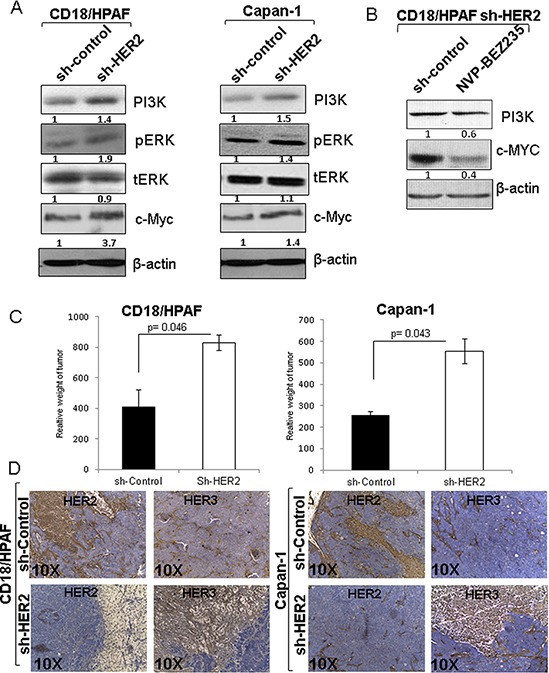
Involvement of PI3K, ERK and c-MYC in HER3/MUC4 mediated signaling in HER2 knockdown pancreatic cancer cells and tumorigenic properties of HER2 knockdown cells **A.** Western blot results show that PI3K, phosphorylation of ERK and c-Myc were elevated in HER2 knockdown cells than control cells. The increased expression and activity of PI3K, ERK and c-MYC may be involve the HER3/MUC4 mediated signaling in HER2 low expressing pancreatic cancer cells. **B.** We have also confirmed the involvement of PI3K and c-Myc by PI3k inhibitor NVP-BEZ235 treatment. This finding suggests that hyperproliferation of HER2 knockdown cells is due to downstream activity of PI3K and c-Myc. **C.** The tumorigenicity assay results indicate that HER2 knockdown pancreatic cancer cells have high tumor weight than control cells injected in mice. **D.** Corresponding immunostaining of HER2 and HER3 in pancreatic cancer cells (CD18/HPAF scrambled control/CD18/HPAF Sh-HER2 and Capan-1 scrambled control/Capan-1 Sh-HER2) subcutaneous tumors, indicates that HER3 expression is increased in HER2 knockdown tumor tissues (figure magnification 10X).

As mentioned earlier, HER3 is overexpressed in the HER2 knockdown cells. Similarly, we have also seen increased expression of phosphatidylinositol 3-kinase (PI3K) in the HER2 knockdown cells compared to scrambled control cells (Figure 5A). Since HER3 has six docking sites for the PI3K [35, 36], overexpression of HER3 could activate PI3K mediated downstream signaling in HER2 knockdown cells. In addition, we observed an increase in the phosphorylation of ERK1/2 and proto-oncogene c-Myc in the HER2 knockdown cells as compared to scrambled control cells (Figure 5A). A previous report has demonstrated that PI3K activates ERK signaling for c-Myc mediated tumor cell growth, proliferation and invasion [37]. Consistent with the previous finding, our data also showed that the association of HER3/MUC4 is accompanied with an activation of PI3K/ERK/c-Myc signaling axis in the HER2 knockdown cells.

### Inhibition of PI3K signaling by NVP-BEZ235 affects c-Myc expression in CD18/HPAF pancreatic cancer cells

In our study, knockdown of HER2 in pancreatic cancer cells showed an enhanced HER3 protein expression, upregulation of PI3K/ERK/C-Myc pathway and subsequent increase in cell proliferation. To understand the direct relationship of PI3K with c-Myc under HER2 suppressed condition, we performed PI3K inhibitor study on CD18/HPAF cells. For that, we chose a dual PI3K and mTOR inhibitor; NVP-BEZ235 to access whether PI3K inhibition affects c-Myc protein level. As expected, we observed a significant reduction of PI3K and c-Myc protein expression level following treatment of the HER2 knockdown cells with NVP-BEZ235 as compared to untreated control cells (Figure 5B). This result provides further evidence supporting a PI3K mediated c-Myc activation in the HER2 knockdown cells.

### Loss of HER2 increased tumorigenicity in pancreatic cancer cells

Since HER2-knockdown resulted in increased proliferative activity in pancreatic cancer cells, we examined the *in vivo* tumorigenic activity of HER2 knockdown pancreatic cancer cells. HER2-knockdown CD18/HPAF and Capan-1 cells and their corresponding scrambled control cells were subcutaneously injected into immunodeficient female nude mice (1 × 10^6^ cells/mice). After 40 days of injection, we observed that mice implanted with the HER2-knockdown cells [CD18/HPAF-Sh-HER2 (*p* = 0.046) and Capan-1-Sh-HER2 (*p* = 0.043)] produced tumors with greater weight compared to mice injected with scrambled control cells (Figure 5C). Further, immunohistochemistry results indicate that HER3 expression was considerably high in the HER2 knockdown tumors (Sh-HER2 CD18/HPAF and Sh-HER2 Capan-1) as compared to scrambled control tumors (Figure 5D). Altogether, these results indicate that HER2-knockdown leads to increased tumorigenic properties.

### HER2 knockdown enhances motile property of pancreatic cancer cells

**Figure 6:**
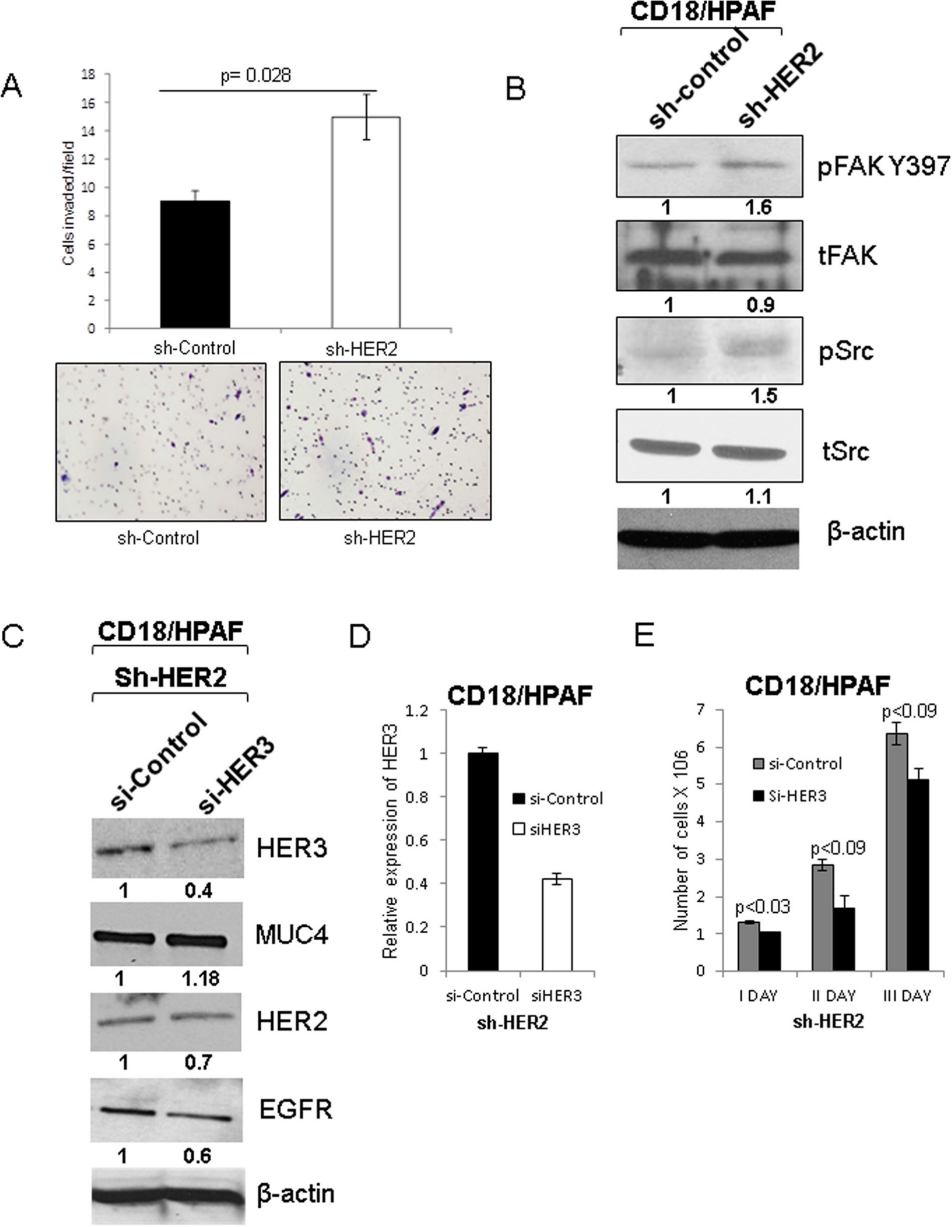
Effect of HER2 knockdown on motile properties of pancreatic cancer cells and transient knockdown of HER3 and its effect on EGFR family proteins and MUC4 **A.** The motile properties of HER2 knockdown cells were significantly increased in comparison to scrambled control cells CD18/HPAF with quantitative data (Figure magnification 20X). **B.** Tyrosine phosphorylation of focal adhesion kinase 397 (pFAK Y397) and phosphorylation of Src were increased in HER2 knockdown CD18/HPAF cells. **C.** Represents the siHER3 transfection in HER2 knockdown pancreatic cancer cells. Upon HER3 transient knockdown in HER2 knockdown (HER-/HER3-) cells, no change in the expression of MUC4 and other EGFR family proteins such as HER2 and EGFR was observed. **D.** The mRNA level of HER3 was analyzed to confirm the HER3 inhibition followed by siRNA treatment. **E.** In addition, cell proliferation assay data indicates that loss of both HER3 and HER2 results in decreased proliferation.

To examine the motile properties of HER2 knockdown cells, we performed cell migration assay. HER2 knockdown CD18/HPAF pancreatic cancer cells have more motile properties than scrambled control cells (*p = 0.028*) (Figure 6A). In support of this process, phosphorylation of Focal adhesion kinase (FAK Y397) and phosphorylation of Src were also observed to be elevated in the HER2 knockdown CD18/HPAF cells than scrambled control cells (Figure 6B), which indicates that the knockdown cells had a higher migration capacity.

### Silencing of HER3 in CD18/HPAF HER2-knockdown cells decrease pancreatic cancer cell proliferation

To further investigate the role of HER3 in the cells lacking HER2, we transiently knock down HER3 in the HER2-knockdown pancreatic cancer cells [HER2 (−) and HER3 (−)]. We observed that loss of HER3 in HER2 knockdown cells did not significantly affect the expression of MUC4 (Figure 6C and Supplementary Figure 4C), and HER3 knockdown was further validated by RT-PCR analysis (Figure 6D). However, loss of both HER2 and HER3 resulted in a decrease in the proliferation of pancreatic cancer cells (Figure 6E), suggesting that deficiency of both HER2 and HER3 affects the pancreatic cancer cell proliferation. In addition, we have also performed siHER3 transfection in scramble control cells (HER2 (−) and HER3 (+)) and did not observe significant difference in HER2 and MUC4 expression (Supplementary Figure 4A). These results indicate that HER2 and HER3 are critical for MUC4-mediated pancreatic cancer cell proliferation.

## DISCUSSION

Amplification and/or overexpression of HER2 have been demonstrated to be involved in the progression and advancement of variety of cancer including pancreatic cancer [11, 24, 38, 39]. Further, its oncogenic function requires interaction with other EGFR family members including HER3 [12, 40]. Studies have also reported that HER2 overexpression was detected in 21–50% of pancreatic cancer tissues and correlated with disease advancement. Similarly, HER3 is also overexpressed and has been associated with poor prognosis of pancreatic cancer [17, 41, 42]. Friess *et al* have reported that HER3 mRNA was 6.7 fold elevated in pancreatic cancer as compared to normal pancreas, which was further confirmed by Immunohistochemistry showing that 47% (27/58) of pancreatic cancer tissues expressed HER3 [16]. Similarly, another study have demonstrated that HER3 is overexpressed in 52 of the 126 tissue of pancreatic cancer samples (41.3%), suggesting the clinical significance of HER3 in pancreatic cancer [17]. Based on these studies, we conclude that HER3 is overexpressed in pancreatic cancer and significantly correlate with the poor prognosis of pancreatic cancer patients.

HER2 and HER3 are overexpressed in pancreatic cancer tissues in an independent manner but their co expression and functions are not understood. In this study, we analyzed the relative expression of HER2 and HER3 in pancreatic cancer tissues as well as in spontaneous mouse model of pancreatic cancer. Our findings suggest that HER3 is strongly overexpressed in pancreatic cancer tissues in relation to HER2, further signifying the role of HER3 in pancreatic cancer. According to the Oncomine database, HER3 (fold change 5.14, *p*-value: 1.26E-4) is significantly overexpressed in pancreatic cancer than HER2 (fold change 3.05, *p*-value: 5.31E-6), which strongly suggests that HER3 has a crucial role in pancreatic cancer. Holbro *et al* found that HER2 alone is not sufficient for cancer cell proliferation, and the cells require the presence of HER3 [12]. Further published reports state that HER3 is essential for pancreatic cancer development and disease progression [17, 18].

Next we wanted to identify the mechanism behind the HER family mediated pancreatic cancer cell growth and metastasis. In the present study, we have identified an alternative mechanism that contributes towards the aggressiveness of pancreatic cancer cells in the absence of HER2. Upon HER2 knockdown in pancreatic cancer cell, we observed an elevated expression of HER3 and MUC4 that leads to increased proliferation of pancreatic cancer cells. Interestingly, we did not observe any significant change in EGFR and HER4 protein levels. An earlier report has demonstrated that when HER2, EGFR and HER4 were inhibited using tyrosine kinase (TK) inhibitors, HER3 was upregulated, and this appeared to facilitate cancer cell survival and proliferation [43]. It was further supported by recent report that, upon TK inhibitor treatment, HER3 is upregulated and its activity was regulated by residual HER2 [44]. These reports and our observations suggest that the loss of HER2 leads to HER3 overexpression, which is required for maintaining the proliferative property of pancreatic cancer cells.

We have previously reported that MUC4 interacts with and stabilizes HER2, which resulted in the proliferation and metastasis of pancreatic and ovarian cancer cells [24, 25]. Also, MUC4 knockdown and overexpression induces changes in the relative levels of HER2. Previous studies have demonstrated that MUC4 is involved in pancreatic cancer cell proliferation and metastasis [27, 45] and it is differentially expressed at different stages of pancreatic cancer [46, 47]. Due to the presence of 3 EGF domains in MUC4, we investigated it can interact with HER3. Our finding suggests that HER3 strongly interacts with MUC4 in HER2 knockdown pancreatic cancer cells as demonstrated by reciprocal immunoprecipitation assay, which suggests that HER3/MUC4 association is essential for HER2 low pancreatic cancer cells. In addition, we observed co-localization of MUC4 and HER3 in HER2 knockdown cells as well as in spontaneous mice tumor tissues (KPC; Kras^G12D^; Trp53^R172H/+^; Pdx-Cre) of pancreatic cancer. Similarly, HER2-dependent breast tumors preferentially associate with HER3 for cancer cell growth and emphasizing the importance of HER2/HER3 association in breast cancer pathogenesis [40]. Further, we have also observed that the adaptor molecule Grb2 interacts with both HER3 and MUC4 in pancreatic cancer cells, which suggests that HER3/MUC4 association leads to recruitment of Grb2 for HER3/MUC4 mediated downstream signaling pathway. Grb2 contains the SH2 domain which facilitates protein-protein interaction through phosphorylated tyrosines [48] as observed in EGFR family members. Grb2 contributes to cancer cell proliferation and migration through RTK pathways [49].

Our functional studies indicate that HER2 knockdown cells have significantly higher growth rate than control cells, which was validated by expression of Cyclins. Similarly, *in vivo* tumorigenic results demonstrated that HER2 knockdown cells have increased tumor growth. These results strongly suggest that increased growth rate of HER2 knockdown cells may be due to HER3/MUC4 signaling. Furthermore, HER2 knockdown pancreatic cancer cells exhibit increased motility as indicated by cell migration assay and it is supported by increased phosphorylation of focal adhesion kinase and Src.

**Figure 7:**
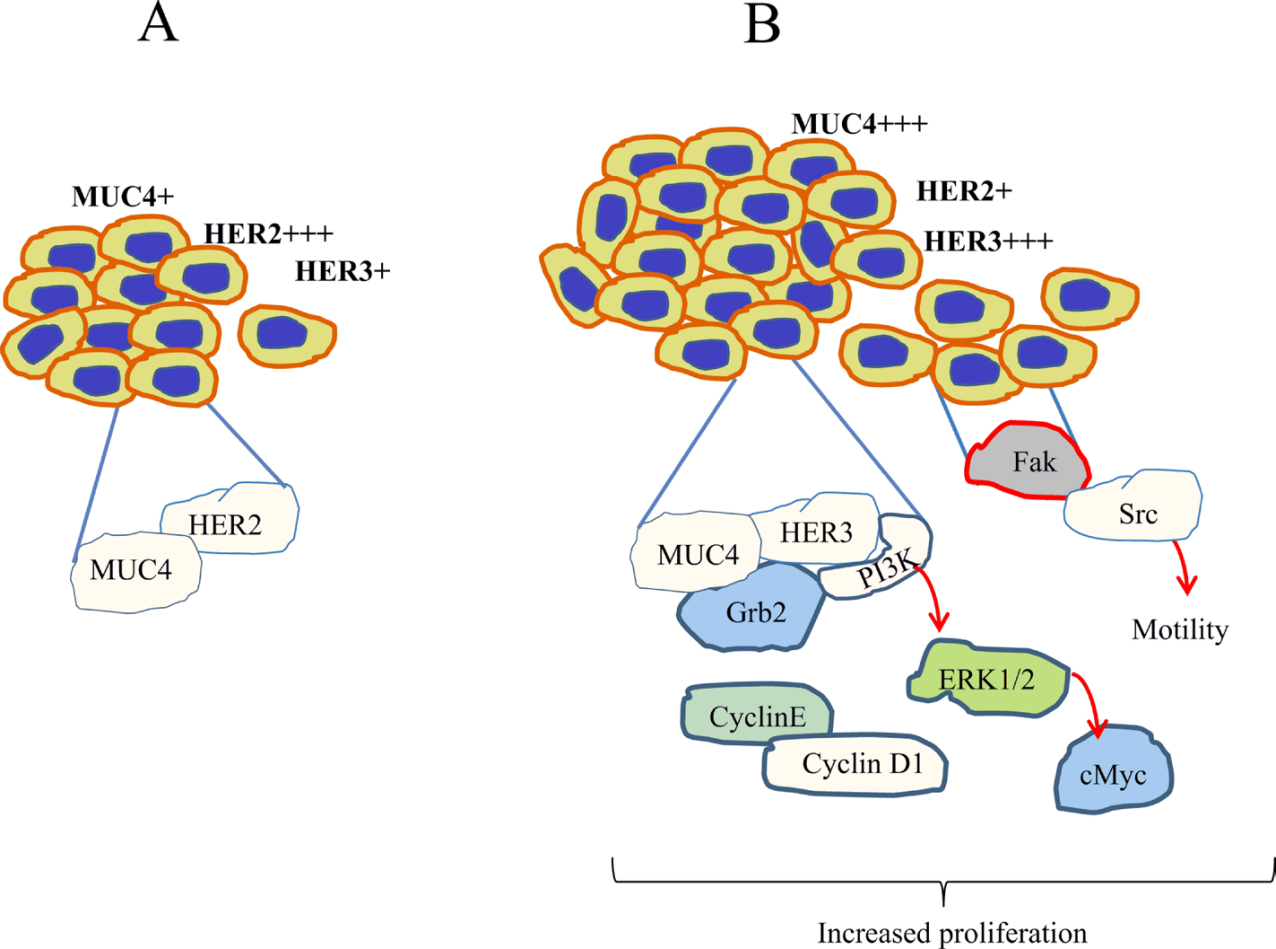
Schematic diagram of HER3/MUC4 mediated pancreatic cancer cell proliferation In HER2 low pancreatic cancer cells [B], HER3 associates with MUC4 that leads to increased PI3K activity that promote cell proliferation than HER2 high pancreatic cancer cells [A]. Further, phosphorylation of ERK and c-Myc are also involved in HER3/MUC4 mediated signaling pathways in HER2 low pancreatic cancer cells. In addition, our studies suggested increased motility due to increased phosphorylation of Focal adhesion kinase and phosphorylation of Src in HER2 low pancreatic cancer cells. Collectively, loss of both HER2 and HER3 leads to decreased proliferation, which suggests that both HER2 and HER3 are involved in MUC4 mediated pancreatic cancer cell proliferation

Next we sought to elucidate the mechanism behind HER3/MUC4 mediated pancreatic cancer cell hyperproliferation in HER2 knockdown cells. We observed elevated expression of PI3K, c-MYC and phosphorylation of ERK in HER2 knockdown cells than control cells, suggesting that HER3/MUC4 mediated hyperproliferation of HER2 low pancreatic cancer cells through the PI3K/ERK/c-Myc pathway. Further, we have shown that inhibition of PI3K using NVP-BEZ235 resulted in decrease in c-Myc protein expression, signifying our observed effects of HER2 knockdown in pancreatic cancer cells. In support our findings, Takikita *et al* had demonstrated HER3 as a critical mediator for cancer cell survival through PI3K pathway in the absence of HER2 oncogenic signaling followed by Tyrosine kinase inhibitor [15, 43]. Collectively, the *in vitro* and *in vivo* results demonstrate a hyperproliferative activity in the HER2 low pancreatic cancer cells through PI3K/ERK/c-Myc axis (Figure 7). In addition, migration of pancreatic cancer cells is significantly increased in HER2 knockdown cells via increased phosphorylation of FAK (Y397) and Src in HER2 knockdown cells. Phosphorylated FAK at Y397 have high binding affinity with Src that mediates downstream signaling for cell migration [50]. This result suggests that high motile properties of HER2 knockdown cells is due to increased phosphorylation of FAK/Src.

To identify the importance of HER3 in pancreatic cancer cell proliferation, we transiently knockdown HER3 in HER2 low pancreatic cancer cells, and found that MUC4 protein expression level did not change significantly in these cells. These results suggest that there may be an interdependent mechanism between HER2 and HER3 for MUC4 mediated pancreatic cancer cell proliferation.

## CONCLUSION

In HER2 low pancreatic cancer cells, HER3 is overexpressed and associates with MUC4 for increased proliferation of pancreatic cancer cells through PI3K/ERK/c-Myc axis (Figure 7). Similarly, motility of pancreatic cancer is increased in HER2 depleted cells through FAK/Src downstream pathway. Further, deficiency of both HER2 and HER3 leads to decreased proliferation of pancreatic cancer cells. Collectively, our results suggest that both HER2 and HER3 are indispensable for MUC4 mediated pancreatic cancer cell proliferation. Based on our studies, we assume that overexpression of HER3 in pancreatic cancer may be the reason behind therapeutic failure of Herceptin. Our study also provides a rationale for combined targeting of HER family proteins to abrogate MUC4 mediated oncogenic signaling for improving pancreatic cancer patient survival.

## MATERIALS AND METHODS

### Cell culture

CD18/HPAF and Capan-1 cells were procured from American Type Culture Collection [ATCC] (Manassas, VA, USA) and cell lines were propagated in a humidified atmosphere containing 5% CO_2_ at 37°C and cultured in Dulbecco's modified Eagle's medium supplemented with 10% fetal calf serum and antibiotics. HER2 was stably knockdown using HER2 sh-RNA (GAAACCTGGAACTCACCTAC) [which target N-terminal portion of HER2], (sh-HER2seq2-GCAGAGGATGGAACACAGCGGTGTGAGAA) [which target C-terminal of HER2] and (sh-HER2seq3-TGTTGGATGATTGACTCTGAATGTCGGCC) [which target C-terminal region of HER2] construct (pSUPER-Retro-sh-HER2) in HER2 expressing CD18/HPAF and Capan-1 cells by stable transfection method. Scramble control (sh-control) and pSUPER-Retro-sh-HER2 has been transfected into packaging cell Phoenix using Lipofectamine 2000 (Invitrogen, Carlsbad, CA, USA). After 48 h post transfection, the viral particles were collected and used to infect CD18/HPAF and Capan-1 cells. Pooled population of HER2 knockdown cells were obtained using antibiotic selection (Puromycin 4 μg/ml), and were further expanded to confluent levels to obtain stably transfected cells. The dual PI3K and mTOR inhibitor (NVP-BEZ235, LC laboratories, MA, USA) was dissolved in dimethyl sulfoxide (DMSO) and prepared as 10mM stock and stored at −20°C as per the manufacturer's recommendation.

### Transient knockdown of HER3 in HER2 knockdown cells

siRNA containing the HER3 target sequence ACCACGGTATCTGGTCATAAA (Thermo Scientific) was synthesized. Oligonucleotides of HER3 scrambled control and siRNA were transfected in to the cell lines using oligofectamine transfection reagent according to manufacturer's instructions. In a six-well plate containing cells, 200 picomoles of HER3 siRNA was used with the transfection reagent [51].

### Immunoblot

Using RIPA buffer (50 mM Tris-HCl, 150 mM NaCl, 1% NP-40, 0.5% sodium deoxycholate and 0.1% SDS) containing protease inhibitors (1 mM phenyl-methyl sulphonyl fluoride, 1 mg/ml aprotinin, 1 mg/ml leupeptin) CD18/HPAF and Capan-1 pancreatic cancer cells were lysed and protein content was quantified. Then samples (20 μg) were separated by 10% SDS-polyacrylamide gel electrophoresis, for MUC4 2% SDS-agarose gel was used. The proteins were transferred to polyvinyldifluoride membranes (Millipore Corporation, Bedford, MA, USA) by electro or capillary transfer for immunodetection. The transferred membrane was washed with PBST [phosphate-buffered saline (PBS) and 0.1% Tween 20], blocked in 5% non-fat dry milk in PBS for at least 1 h and then incubated with primary antibodies: HER2 (Rabbit, 1:1000, Cell Signaling), MUC4 (8G7) (Mouse, 1:1000), HER3 (Rabbit, 1:1000, Cell Signaling), EGFR (rabbit, 1:1000, 1C1), pEGFR-Y-1068 (rabbit, 1:1000, Cell Signaling), PI3K- p85 subunit rabbit, 1:2000, cell signaling, c-Myc (Mouse 1:1000, Santa Cruz biotechnology), Grb2 (rabbit, 1:2000, Cell Signaling), Cyclin D1 (rabbit, 1:1500, Santa Cruz biotechnology), Cyclin A (Mouse, 1:1500, Santa Cruz biotechnology), and anti-β-actin (mouse 1:500) (diluted in 2% bovine serum albumin in PBS) overnight at 4°C. Then the membranes were washed (3 × 10min) in PBST at room temperature and probed with the appropriate secondary antibodies at 1:5000 dilutions for 1 h at room temperature and washed 3× 10min with PBST. The signal was detected with the ECL chemiluminescence kit (Amersham Bioscience, Amersham Place Little Chalfont, Buckinghamshire, UK).

### Immunoprecipitation analysis

HER2, HER3, Grb2 and MUC4 antibodies were incubated overnight with CD18/HPAF cell lysates (500 μg) in a 750μl total volume. Protein A+G-Sepharose beads (Sigma-Aldrich Corp., St Louis, MO, USA) were added to the lysate-antibody mix and incubated on a rotating platform for 4 h at 4°C and then washed four times with lysis buffer. To ensure the MUC4 and HER3 interactions, we have also done immunodepletion of HER2 in HER2 knockdown cells and then immunoprecipitate with HER3 antibody. The immunoprecipitates and total cell lysates were electrophoretically resolved on SDS-polyacrylamide gel electrophoresis (8%). Resolved proteins were transferred onto the polyvinyldifluoride membrane. The membranes were blocked in 5% non-fat dry milk in PBS for at least 1 h and then incubated with primary antibodies (anti-HER2, anti-HER3, anti-Grb2 and anti-MUC4). The immunoblots were washed five times (5 × 10 min) with PBST, incubated for 1 h with respective secondary antibodies, washed five times (5 × 10 min) with PBST, reacted with enhanced chemiluminescence ECL reagent (Amersham Biosciences) and exposed to X-ray film to detect the signal.

### Confocal microscopy analysis

CD18/HPAF and Capan-1 cells were grown on autoclaved cover slips for 48 h and incubated with Hanks buffer [0.1M HEPES] for 15 min. Cells were then fixed with 100% methanol at −20°C for 2 min and washed with PBS. 10% goat serum (Jackson Immunoresearch Labs, Inc., West Grove, PA, USA) was used for blocking the cells for at least 30 min, followed by overnight incubation with primary antibodies; HER2 (rabbit), HER3 (rabbit) and MUC4 (mouse) (8G7) at 4°C. After primary antibody incubation, cells were washed with PBS [3 × 5 min] and incubated with fluorescein isothiocyanate-conjugated antimouse and Texas red-conjugated anti-rabbit secondary antibodies (Jackson Immunoresearch labs, Inc.) for 30 min at room temperature in the dark. DAPI [4′, 6-Diamidino-2-Phenylindole, Dihydrochloride] was used for nuclear staining. Cells were washed again (5 × 5 min) and mounted on glass slides in anti-fade vectashield mounting medium (Vector Laboratories, Burlingame, CA, USA). Laser confocal microscopy was performed using an LSM 510 microscope (Carl Zeiss GmbH, Jena, Germany). For Immunohistochemistry, the slides were processed for immunostaining as described previously [29]. Composite score of tissue staining were transformed into heap map for better visualization.

### Cell motility assay

Motility assay was performed by using a chamber containing polyethylene teraphthalate membranes (six-well insert, pore size of 8 μm; Becton Dickinson, Franklin Lakes, NJ, USA). The pancreatic cancer cells were seeded at 1 × 10^6^ in serum free media. After 24 h, migrated cells that had reached the lower chamber (serum containing media) were stained with Quick-Diff kit staining solution and the migrated cells were counted in 8 different random fields and the average number of motile cells per representative field was calculated.

### Cell cycle analysis by double thymidine block method

For cell cycle experiments, pancreatic cancer cells [Scrambled control & Sh-HER2-CD18/HPAF and Scrambled control & Sh-HER2-Capan-1] were seeded at 1 × 10^6^ in 100 mm petridish. Thymidine (Sigma, St. Louis, Missouri, USA) was added to culture medium at concentration of 2 mM for 12 h for synchronize the cells. Following two washes with serum-free media, the cells were released from the thymidine block by culturing in fresh medium containing 24 mM of 2′-Deoxycytidine. After 9 h of incubation, a second thymidine block was initiated and completed after 14 h. Then release the cells from the thymidine block by washing in phosphate buffered saline and replaced with complete culture medium. Then cells were fixed in 70% ethanol. After fixation, the cells were stained with Telford's reagent (90 mM EDTA, 2.5 mU of RNase A/ml, 50 mg of propidium iodide/ml and 0.1% Triton X-100 in PBS). Then, the total DNA content was analyzed using the fluorescence-activated cell sorting method. Similarly, growth kinetic assay was performed as described earlier [45].

### Inhibitor treatment for PI3K

For drug treatment studies, CD18/HPAF pancreatic cancer cells were grown in 60 mm dishes supplemented with complete medium. The PI3k inhibitor NVP-BEZ235 was dissolved in DMSO and incubated with pancreatic cancer cells at 1 μM concentration for 24 h. We choose this concentration based upon the previous report [52].

### *In vivo* tumorigenicity assay

To demonstrate the *in vivo* tumorigenicity of HER2 knockdown cells, a tumor xenograft model was created by subcutaneous implantation of HER2 knockdown CD18/HPAF and Capan-1 pancreatic cancer cells (1 × 10^6^cells/mice) in 4–6 weeks old female athymic nude mice. During the course of *in vivo* tumorigenicity assay the mice were fed with *ad libitum* food and water and were maintained on a 12 h dark/light cycle. The *in vivo* examination was conducted according to the UNMC Institutional Animal Care and Use Committee (IACUC) guidelines. Prior to the subcutaneous injection of human pancreatic cancer cells all the mice were weighed and randomized into four groups. Groups 1 and 2 (*n* = 4 per group) received implantation of scrambled control and HER2 stable knockdown CD18/HPAF cells. Similarly, groups 3 and 4 (*n* = 4 per group) received scrambled control and HER2 knockdown Capan-1 cells. After 40 days of subcutaneous injection of pancreatic cancer cells, the animals were euthanized and the tumors were excised and weighed.

### Generation of tumor tissues from genetically engineered KPC mouse model

The KPC (Kras^G12D^; Trp53^R172H/+^; Pdx-1-Cre) mouse model was developed by David Tuveson (53) and maintained at UNMC by crossing LSL-Kras^G12D^ with LSL-Trp53^R172H/+^ transgenic mice as described previously [28, 54]. Tissues from the triple transgenic animals (Kras^G12D^; Trp53^R172H/+^; Pdx-1-Cre) and their contemporary littermates harvested at 10, 20 and 25 weeks of age were used for immunohistochemical analysis.

## SUPPLEMENTARY FIGURES



## References

[1] Baselga J, Swain SM (2009). Novel anticancer targets: revisiting ERBB2 and discovering ERBB3. Nat Rev Cancer.

[2] Citri A, Yarden Y (2006). EGF-ERBB signalling: towards the systems level. Nat Rev Mol Cell Biol.

[3] Pinkas-Kramarski R, Soussan L, Waterman H, Levkowitz G, Alroy I, Klapper L, Lavi S, Seger R, Ratzkin BJ, Sela M (1996). Diversification of Neu differentiation factor and epidermal growth factor signaling by combinatorial receptor interactions. EMBO J.

[4] Burgess AW, Cho HS, Eigenbrot C, Ferguson KM, Garrett TP, Leahy DJ, Lemmon MA, Sliwkowski MX, Ward CW, Yokoyama S (2003). An open-and-shut case? Recent insights into the activation of EGF/ErbB receptors. Mol Cell.

[5] Mahipal A, Mcdonald MJ, Witkiewicz A, Carr BI (2012). Cell membrane and cytoplasmic epidermal growth factor receptor expression in pancreatic ductal adenocarcinoma. Med Oncol.

[6] Valsecchi ME, McDonald M, Brody JR, Hyslop T, Freydin B, Yeo CJ, Solomides C, Peiper SC, Witkiewicz AK (2012). Epidermal growth factor receptor and insulinlike growth factor 1 receptor expression predict poor survival in pancreatic ductal adenocarcinoma. Cancer.

[7] Day JD, Digiuseppe JA, Yeo C, Lai-Goldman M, Anderson SM, Goodman SN, Kern SE, Hruban RH (1996). Immunohistochemical evaluation of HER-2/neu expression in pancreatic adenocarcinoma and pancreatic intraepithelial neoplasms. Hum Pathol.

[8] Pryczynicz A, Guzinska-Ustymowicz K, Kemona A, Czyzewska J (2008). Expression of EGF and EGFR strongly correlates with metastasis of pancreatic ductal carcinoma. Anticancer Res.

[9] Olayioye MA, Neve RM, Lane HA, Hynes NE (2000). The ErbB signaling network: receptor heterodimerization in development and cancer. EMBO J.

[10] Sithanandam G, Anderson LM (2008). The ERBB3 receptor in cancer and cancer gene therapy. Cancer Gene Ther.

[11] Owens MA, Horten BC, Da Silva MM (2004). HER2 amplification ratios by fluorescence in situ hybridization and correlation with immunohistochemistry in a cohort of 6556 breast cancer tissues. Clin Breast Cancer.

[12] Holbro T, Beerli RR, Maurer F, Koziczak M, Barbas CF, Hynes NE (2003). The ErbB2/ErbB3 heterodimer functions as an oncogenic unit: ErbB2 requires ErbB3 to drive breast tumor cell proliferation. Proc Natl Acad Sci U S A.

[13] Vaught DB, Stanford JC, Young C, Hicks DJ, Wheeler F, Rinehart C, Sanchez V, Koland J, Muller WJ, Arteaga CL (2012). HER3 is required for HER2-induced preneoplastic changes to the breast epithelium and tumor formation. Cancer Res.

[14] Hynes NE, Lane HA (2005). ERBB receptors and cancer: the complexity of targeted inhibitors. Nat Rev Cancer.

[15] Takikita M, Xie R, Chung JY, Cho H, Ylaya K, Hong SM, Moskaluk CA, Hewitt SM (2011). Membranous expression of Her3 is associated with a decreased survival in head and neck squamous cell carcinoma. J Transl Med.

[16] Friess H, Yamanaka Y, Kobrin MS, Do DA, Buchler MW (1995). Enhanced erbB-3 expression in human pancreatic cancer correlates with tumor progression. Clin Cancer Res.

[17] Hirakawa T, Nakata B, Amano R, Kimura K, Shimizu S, Ohira G, Yamada N, Ohira M, Hirakawa K (2011). HER3 overexpression as an independent indicator of poor prognosis for patients with curatively resected pancreatic cancer. Oncology.

[18] Liles JS, Arnoletti JP, Tzeng CW, Howard JH, Kossenkov AV, Kulesza P, Heslin MJ, Frolov A (2010). ErbB3 expression promotes tumorigenesis in pancreatic adenocarcinoma. Cancer Biol Ther.

[19] Gaborit N, Abdul-Hai A, Mancini M, Lindzen M, Lavi S, Leitner O, Mounier L, Chentouf M, Dunoyer S, Ghosh M (2015). Examination of HER3 targeting in cancer using monoclonal antibodies. Proc Natl Acad Sci U S A.

[20] Gala K, Chandarlapaty S (2014). Molecular pathways: HER3 targeted therapy. Clin Cancer Res.

[21] Yamazaki T, Zaal K, Hailey D, Presley J, Lippincott-Schwartz J, Samelson LE (2002). Role of Grb2 in EGF-stimulated EGFR internalization. J Cell Sci.

[22] Sastry L, Cao T, King CR (1997). Multiple Grb2-protein complexes in human cancer cells. Int J Cancer.

[23] Jaulin-Bastard F, Saito H, Le BA, Ollendorff V, Marchetto S, Birnbaum D, Borg JP (2001). The ERBB2/HER2 receptor differentially interacts with ERBIN and PICK1 PSD-95/DLG/ZO-1 domain proteins. J Biol Chem.

[24] Chaturvedi P, Singh AP, Chakraborty S, Chauhan SC, Bafna S, Meza JL, Singh PK, Hollingsworth MA, Mehta PP, Batra SK (2008). MUC4 mucin interacts with and stabilizes the HER2 oncoprotein in human pancreatic cancer cells. Cancer Res.

[25] Ponnusamy MP, Seshacharyulu P, Vaz A, Dey P, Batra SK (2011). MUC4 stabilizes HER2 expression and maintains the cancer stem cell population in ovarian cancer cells. J Ovarian Res.

[26] Andrianifahanana M, Moniaux N, Schmied BM, Ringel J, Friess H, Hollingsworth MA, Buchler MW, Aubert JP, Batra SK (2001). Mucin (MUC) gene expression in human pancreatic adenocarcinoma and chronic pancreatitis: a potential role of MUC4 as a tumor marker of diagnostic significance. Clin Cancer Res.

[27] Chaturvedi P, Singh AP, Moniaux N, Senapati S, Chakraborty S, Meza JL, Batra SK (2007). MUC4 mucin potentiates pancreatic tumor cell proliferation, survival, and invasive properties and interferes with its interaction to extracellular matrix proteins. Mol Cancer Res.

[28] Rachagani S, Torres MP, Kumar S, Haridas D, Baine M, Macha MA, Kaur S, Ponnusamy MP, Dey P, Seshacharyulu P (2012). Mucin (Muc) expression during pancreatic cancer progression in spontaneous mouse model: potential implications for diagnosis and therapy. J Hematol Oncol.

[29] Moniaux N, Chaturvedi P, Varshney GC, Meza JL, Rodriguez-Sierra JF, Aubert JP, Batra SK (2007). Human MUC4 mucin induces ultra-structural changes and tumorigenicity in pancreatic cancer cells. Br J Cancer.

[30] Tzahar E, Waterman H, Chen X, Levkowitz G, Karunagaran D, Lavi S, Ratzkin BJ, Yarden Y (1996). A hierarchical network of interreceptor interactions determines signal transduction by Neu differentiation factor/neuregulin and epidermal growth factor. Mol Cell Biol.

[31] Cho HS, Leahy DJ (2002). Structure of the extracellular region of HER3 reveals an interdomain tether. Science.

[32] Guda C, King BR, Pal LR, Guda P (2009). A top-down approach to infer and compare domain-domain interactions across eight model organisms. PLoS One.

[33] Punta M, Coggill PC, Eberhardt RY, Mistry J, Tate J, Boursnell C, Pang N, Forslund K, Ceric G, Clements J (2012). The Pfam protein families database. Nucleic Acids Res.

[34] Lane HA, Beuvink I, Motoyama AB, Daly JM, Neve RM, Hynes NE (2000). ErbB2 potentiates breast tumor proliferation through modulation of p27(Kip1)-Cdk2 complex formation: receptor overexpression does not determine growth dependency. Mol Cell Biol.

[35] Fedi P, Pierce JH, Di Fiore PP, Kraus MH (1994). Efficient coupling with phosphatidylinositol 3-kinase, but not phospholipase C gamma or GTPase-activating protein, distinguishes ErbB-3 signaling from that of other ErbB/EGFR family members. Mol Cell Biol.

[36] Prigent SA, Gullick WJ (1994). Identification of c-erbB-3 binding sites for phosphatidylinositol 3′-kinase and SHC using an EGF receptor/c-erbB-3 chimera. EMBO J.

[37] Tsai WB, Aiba I, Long Y, Lin HK, Feun L, Savaraj N, Kuo MT (2012). Activation of Ras/PI3K/ERK pathway induces c-Myc stabilization to upregulate argininosuccinate synthetase, leading to arginine deiminase resistance in melanoma cells. Cancer Res.

[38] Agus DB, Akita RW, Fox WD, Lewis GD, Higgins B, Pisacane PI, Lofgren JA, Tindell C, Evans DP, Maiese K (2002). Targeting ligand-activated ErbB2 signaling inhibits breast and prostate tumor growth. Cancer Cell.

[39] Franklin MC, Carey KD, Vajdos FF, Leahy DJ, de Vos AM, Sliwkowski MX (2004). Insights into ErbB signaling from the structure of the ErbB2-pertuzumab complex. Cancer Cell.

[40] Lee-Hoeflich ST, Crocker L, Yao E, Pham T, Munroe X, Hoeflich KP, Sliwkowski MX, Stern HM (2008). A central role for HER3 in HER2-amplified breast cancer: implications for targeted therapy. Cancer Res.

[41] Ocana A, Vera-Badillo F, Seruga B, Templeton A, Pandiella A, Amir E (2013). HER3 overexpression and survival in solid tumors: a meta-analysis. J Natl Cancer Inst.

[42] Thomas G, Chardes T, Gaborit N, Mollevi C, Leconet W, Robert B, Radosevic-Robin N, Penault-Llorca F, Gongora C, Colombo PE (2014). HER3 as biomarker and therapeutic target in pancreatic cancer: new insights in pertuzumab therapy in preclinical models. Oncotarget.

[43] Sergina NV, Rausch M, Wang D, Blair J, Hann B, Shokat KM, Moasser MM (2007). Escape from HER-family tyrosine kinase inhibitor therapy by the kinase-inactive HER3. Nature.

[44] Garrett JT, Olivares MG, Rinehart C, Granja-Ingram ND, Sanchez V, Chakrabarty A, Dave B, Cook RS, Pao W, McKinely E (2011). Transcriptional and posttranslational up-regulation of HER3 (ErbB3) compensates for inhibition of the HER2 tyrosine kinase. Proc Natl Acad Sci U S A.

[45] Singh AP, Moniaux N, Chauhan SC, Meza JL, Batra SK (2004). Inhibition of MUC4 expression suppresses pancreatic tumor cell growth and metastasis. Cancer Res.

[46] Swartz MJ, Batra SK, Varshney GC, Hollingsworth MA, Yeo CJ, Cameron JL, Wilentz RE, Hruban RH, Argani P (2002). MUC4 expression increases progressively in pancreatic intraepithelial neoplasia. Am J Clin Pathol.

[47] Zhang J, Zhang X, Zhu Y, Chen Z, Xu Z, Miao Y (2010). Transcriptional regulation of human mucin gene MUC4 in pancreatic cancer cells. Mol Biol Rep.

[48] Matuoka K, Shibata M, Yamakawa A, Takenawa T (1992). Cloning of ASH, a ubiquitous protein composed of one Src homology region (SH) 2 and two SH3 domains, from human and rat cDNA libraries. Proc Natl Acad Sci U S A.

[49] Zhang JS, Koenig A, Young C, Billadeau DD (2011). GRB2 couples RhoU to epidermal growth factor receptor signaling and cell migration. Mol Biol Cell.

[50] Schaller MD, Hildebrand JD, Shannon JD, Fox JW, Vines RR, Parsons JT (1994). Autophosphorylation of the focal adhesion kinase, pp125FAK, directs SH2-dependent binding of pp60src. Mol Cell Biol.

[51] Wang SE, Xiang B, Guix M, Olivares MG, Parker J, Chung CH, Pandiella A, Arteaga CL (2008). Transforming growth factor beta engages TACE and ErbB3 to activate phosphatidylinositol-3 kinase/Akt in ErbB2-overexpressing breast cancer and desensitizes cells to trastuzumab. Mol Cell Biol.

[52] Marone R, Erhart D, Mertz AC, Bohnacker T, Schnell C, Cmiljanovic V, Stauffer F, Garcia-Echeverria C, Giese B, Maira SM (2009). Targeting melanoma with dual phosphoinositide 3-kinase/mammalian target of rapamycin inhibitors. Mol Cancer Res.

[53] Hingorani SR, Wang L, Multani AS, Combs C, Deramaudt TB, Hruban RH, Rustgi AK, Chang S, Tuveson DA (2005). Trp53R172H and KrasG12D cooperate to promote chromosomal instability and widely metastatic pancreatic ductal adenocarcinoma in mice. Cancer Cell.

[54] Vaz AP, Ponnusamy MP, Rachagani S, Dey P, Ganti AK, Batra SK (2014). Novel role of pancreatic differentiation 2 in facilitating self-renewal and drug resistance of pancreatic cancer stem cells. Br J Cancer.

