# Supplementary material for: Novel HER3/MUC4 oncogenic signaling aggravates the tumorigenic phenotypes of pancreatic cancer cells
Source: Oncotarget. 2015 May 16;6(25):21085–99. doi: 10.18632/oncotarget.3912 (PMC4673252; doi:10.18632/oncotarget.3912)
Supplement: Supplementary file 1 [file oncotarget-06-21085-s001.pdf]

SUPPLEMENTARY FIGURES

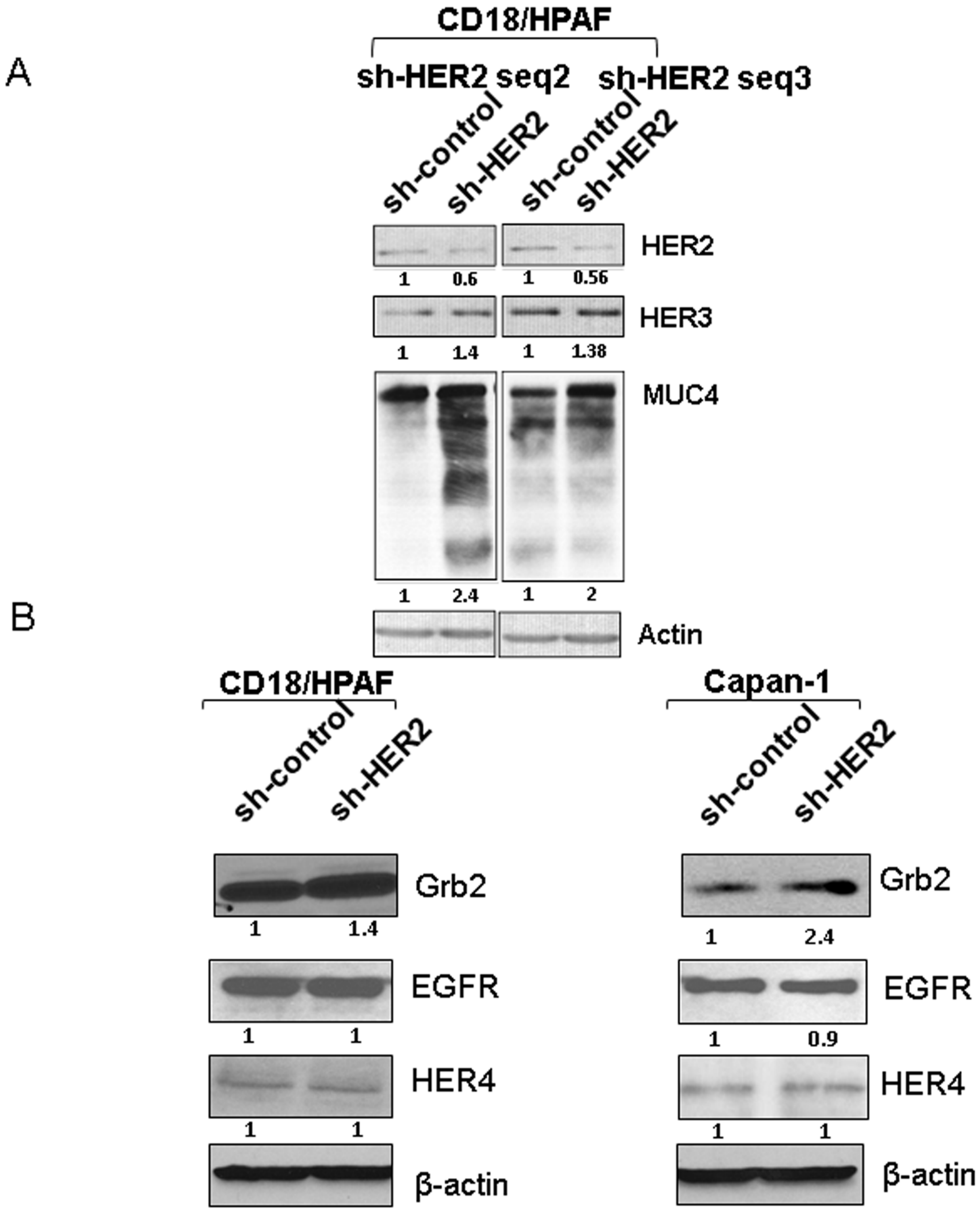

Supplementary Figure S1: Effect of HER2 knockdown on expression of Grb2, EGFR and HER4. A. Represents upregulation of MUC4 and HER3 in HER2 knockdown (sh-HER2 seq-2 and Seq-3) pancreatic cancer cells CD18/HPAF. **B.** As a result of HER2 silencing, Grb2 levels significantly increased as shown in immunoblot studies using two stable knockdown (~40%) pancreatic cancer cells (CD18/HPAF and Capan-1) when compared to scrambled control cells. There was no change in EGFR and HER4 levels in both the pancreatic cancer cells.

Range 1: 22 to 997 Graphics ▼ Next Match ▲ Previous Match

Score	Expect	Method	Identities	Positives	Gaps
900 bits(2327)	0.0	Compositional matrix adjust.	465/983(47%)	621/983(63%)	29/983(2%)

```
Query  25   SQAVCPGTLNGLSVTGDAENQYQTLYKLYERCEVVMGNLEIVLTGHNADLSFLQWIREVT  84
            S  VC GT   L +    E     L  LY+ C+VV GNLE+     NA LSFLQ I+EV
Sbjct  22   STQVCTGTDMKLRLPASPETHLDMLRHLYQGCQVVQGNLELTYLPTNASLSFLQDIQEVQ  81

Query  85   GYVLVAMNEFSTLPLPNLRVVRGTQVYDGKFAIFVM-----LNYNTNSSHA----LRQLR  135
            GYVL+A N+   +PL  LR+VRGTQ+++  +A+ V+     LN  T  + A    LR+L+
Sbjct  82   GYVLIAHNQVRQVPLQRLRIVRGTQLFEDNYALAVLDNGDPLNNTTPVTGASPGGLRELQ  141

Query  136  LTQLTEILSGGVYIEKNDKLCHMDTIDWRDIVRDRDA---EIVVKDNGRSCPPCHEVCKG  192
            L  LTEIL GGV I++N +LC+ DTI W+DI    +     ++  +  R+C PC  +CKG
Sbjct  142  LRSLTEILKGGVLIQRNPQLCYQDTILWKDIFHKNNQLALTLIDTNRSRACHPCSPMCKG  201

Query  193  -RCWGPGSEDCQTLTKTICAPQCNGHCFGPNPNQCCHDECAGGCSGPQDTDCFACRHFND  251
             RCWG  SEDCQ+LT+T+CA  C   C GP P  CCH++CA GC+GP+ +DC AC HFN
Sbjct  202  SRCWGESSEDCQSLTRTVCAGGC-ARCKGPLPTDCCHEQCAAGCTGPKHSDCLACLHFNH  260

Query  252  SGACVPRCPQPLVYNKLTFQLEPNPHTKYQYGGVCVASCPHNFV-VDQTSCVRACPPDKM  310
            SG C   CP  + YN  TF+  PNP  +Y +G  CV +CP+N++  D  SC   CP
Sbjct  261  SGICELHCPALVTYNTDTFESMPNPEGRYTFGASCVTACPYNYLSTDVGSCTLVCPLHNQ  320

Query  311  EVD-KNGLKMCEPCGGLCPKACEGTGSG--SRFQTVDSSNIDGFVNCTKILGNLDFLITG  367
            EV  ++G + CE C   C + C G G       + V S+NI  F  C KI G+L FL
Sbjct  321  EVTAEDGTQRCEKCSKPCARVCYGLGMEHLREVRAVTSANIQEFAGCKKIFGSLAFLPES  380

Query  368  LNGDPWHKIPALDPEKLNVFRTVREITGYLNIQSWPPHMHNFSVFSNLTTIGGRSLYNRG  427
             +GDP      L PE+L VF T+ EITGYL I +WP  + + SVF NL  I GR L+N
Sbjct  381  FDGDPASNTAPLQPEQLQVFETLEEITGYLYISAWPDSLPDLSVFQNLQVIRGRILHNGA  440

Query  428  FSLLIMKNLNVTSLGFRSLKEISAGRIYISANRQLCYHHSLNWTKVLRGPTEERLDIKHN  487
            +SL  ++ L ++ LG RSL+E+ +G   I  N  LC+ H++ W ++ R P +  L    N
Sbjct  441  YSL-TLQGLGISWLGLRSLRELGSGLALIHHNTHLCFVHTVPWDQLFRNPHQALLHTA-N  498

Query  488  RPRRDCVAEGKVCDPLCSSGGCWGPGPGQCLSCRNYSRGGVCVTHCNFLNGEPREFAHEA  547
            RP  +CV EG  C  LC+ G CWGPGP QC++C  + RG  CV  C  L G PRE+ +
Sbjct  499  RPEDECVGEGLACHQLCARGHCWGPGPTQCVNCSQFLRGQECVEECRVLQGLPREYVNAR  558

Query  548  ECFSCHPECQPMEGTATCNGSGSDTCAQCAHFRDGPHCVSSCPHGVLG--AKGPIYKYPD  605
             C  CHPECQP  G+ TC G  +D C  CAH++D P CV+ CP GV    +  PI+K+PD
Sbjct  559  HCLPCHPECQPQNGSVTCFGPEADQCVACAHYKDPPFCVARCPSGVKPDLSYMPIWKFPD  618

Query  606  VQNECRPCHENCTQGCKGPELQDCLGQTLVLIGKTHLTMALTVIAGLVVIFMMLGGTFLY  665
             +  C+PC  NCT  C    + + C  +        + LT  ++ + G++++ ++     +
Sbjct  619  EEGACQPCPINCTHSCVDLDDKGCPAEQRA----SPLTSIISAVVGILLVVVLGVVFGIL  674

Query  666  WRGRRIQ-NKRAMRRYLERGESIEPLDPS-EKANKVLARIFKETELRKLKVLGSGVFGTV  723
             + R+ +  K  MRR L+  E +EPL PS    N+   RI KETELRK+KVLGSG FGTV
Sbjct  675  IKRRQQKIRKYTMRRLLQETELVEPLTPSGAMPNQAQMRILKETELRKVKVLGSGAFGTV  734

Query  724  HKGVWIPEGESIKIPVCIKVIEDKSGRQSFQAVTDHMLAIGSLDHAHIVRLLGLCPGSSL  783
            +KG+WIP+GE++KIPV IKV+ + +  ++ + + D    +  +   ++ RLLG+C  S++
Sbjct  735  YKGIWIPDGENVKIPVAIKVLRENTSPKANKEILDEAYVMAGVGSPYVSRLLGICLTSTV  794

Query  784  QLVTQYLPLGSLLDHVRQHRGALGPQLLLNWGVQIAKGMYYLEEHGMVHRNLAARNVLLK  843
            QLVTQ +P G LLDHVR++RG LG Q LLNW +QIAKGM YLE+  +VHR+LAARNVL+K
Sbjct  795  QLVTQLMPYGCLLDHVRENRGRLGSQDLLNWCMQIAKGMSYLEDVRLVHRDLAARNVLVK  854

Query  844  SPSQVQVADFGVADLLPPDDKQLLYSEAKTPIKWMALESIHFGKYTHQSDVWSYGVTVWE  903
            SP+ V++ DFG+A LL  D+ +      K PIKWMALESI   ++THQSDVWSYGVTVWE
Sbjct  855  SPNHVKITDFGLARLLDIDETEYHADGGKVPIKWMALESILRRRFTHQSDVWSYGVTVWE  914

Query  904  LMTFGAEPYAGLRLAEVPDLLEKGERLAQPQICTIDVYMVMVKCWMIDENIRPTFKELAN  963
            LMTFGA+PY G+   E+PDLLEKGERL QP ICTIDVYM+MVKCWMID   RP F+EL +
Sbjct  915  LMTFGAKPYDGIPAREIPDLLEKGERLPQPPICTIDVYMIMVKCWMIDSECRPRFRELVS  974

Query  964  EFTRMARDPPRYLVIKRES-GPG  985
            EF+RMARDP R++VI+ E  GP
Sbjct  975  EFSRMARDPQRFVVIQNEDLGPA  997
```

HER2

Sequence search results

Show the detailed description of this results page.

We found **6** Pfam-A matches to your search sequence (**5** significant and **1** insignificant). You did not choose to search for Pfam-B matches.

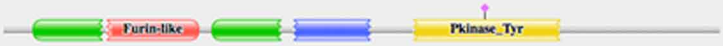

Show the search options and sequence that you submitted.

Return to the search form to look for Pfam domains on a new sequence.

HER3

Sequence search results

Show the detailed description of this results page.

We found **5** Pfam-A matches to your search sequence (**all** significant). You did not choose to search for Pfam-B matches.

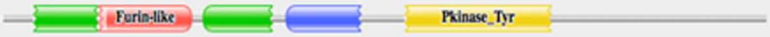

Show the search options and sequence that you submitted.

Return to the search form to look for Pfam domains on a new sequence.

Significant Pfam-A Matches

Show or hide all alignments.

Family	Description	Entry type	Clan	Envelope		Alignment		HMM		HMM length	Bit score	E-value	Predicted active sites	Show/hide alignment
				Start	End	Start	End	From	To					
Recep_L_domain	Receptor L domain	Domain	n/a	55	167	56	166	2	111	112	104.3	3.4e-30	n/a	Show
Furin-like	Furin-like cysteine rich region	Domain	CL0547	169	332	182	332	11	149	149	108.0	3e-31	n/a	Show
Recep_L_domain	Receptor L domain	Domain	n/a	353	474	353	472	1	110	112	102.4	1.3e-29	n/a	Show
GF_recep_IV	Growth factor receptor domain IV	Domain	CL0547	499	630	499	629	1	131	132	162.4	3.7e-48	n/a	Show
Pkinase_Tyr	Protein tyrosine kinase	Domain	CL0016	709	965	710	964	2	258	259	259.4	2.5e-77	n/a	Show

Supplementary Figure S2A and S2B: Bioinformatic prediction of MUC4 and HER3 interaction. S2A. Sequence alignment between Her2 and Her3 shows 47% identity and 63% positives suggesting a close relationship between these two sequences. **S2B.** Domain structure analysis of Her2 and Her3 also reveals almost identical structure indicating functional similarity between these two proteins.

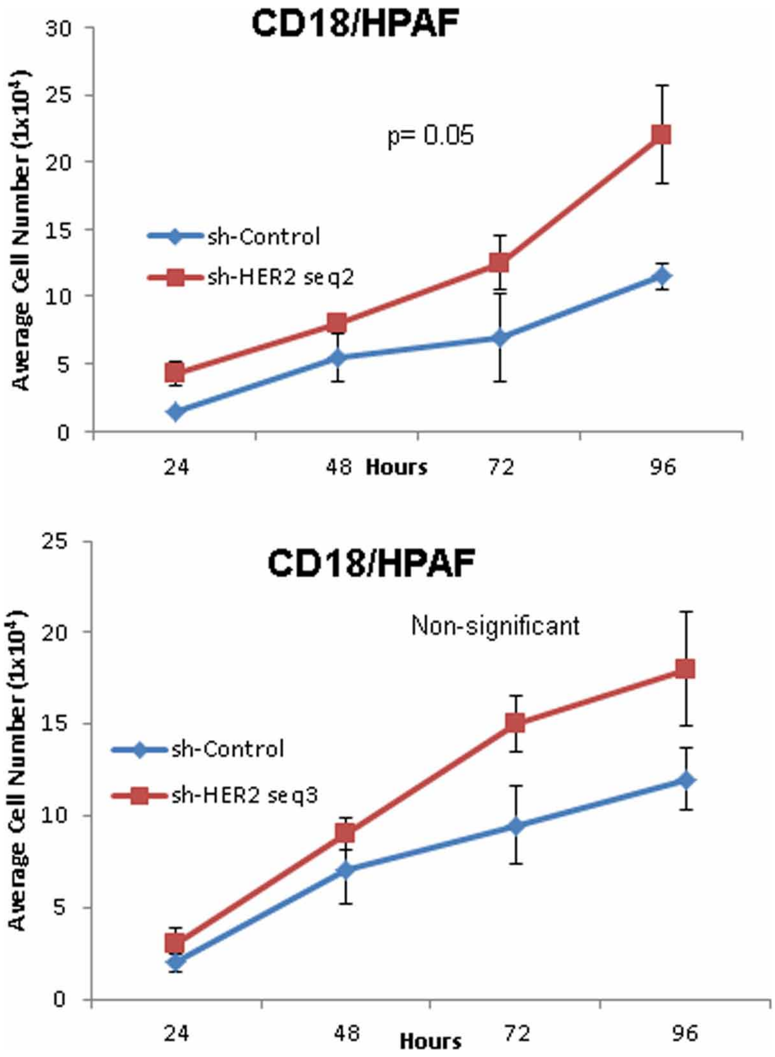

Supplementary Figure S3A and S3B: Effect of HER2 knockdown (two other shHER2) on growth rate of pancreatic cancer cells. In order to validate the HER2 knockdown studies, we utilized the other two shHER2 sequence for growth kinetic assay. As a result of HER2 knockdown we also observed increased proliferation in HER2 knockdown cells in comparison to scramble control cells.

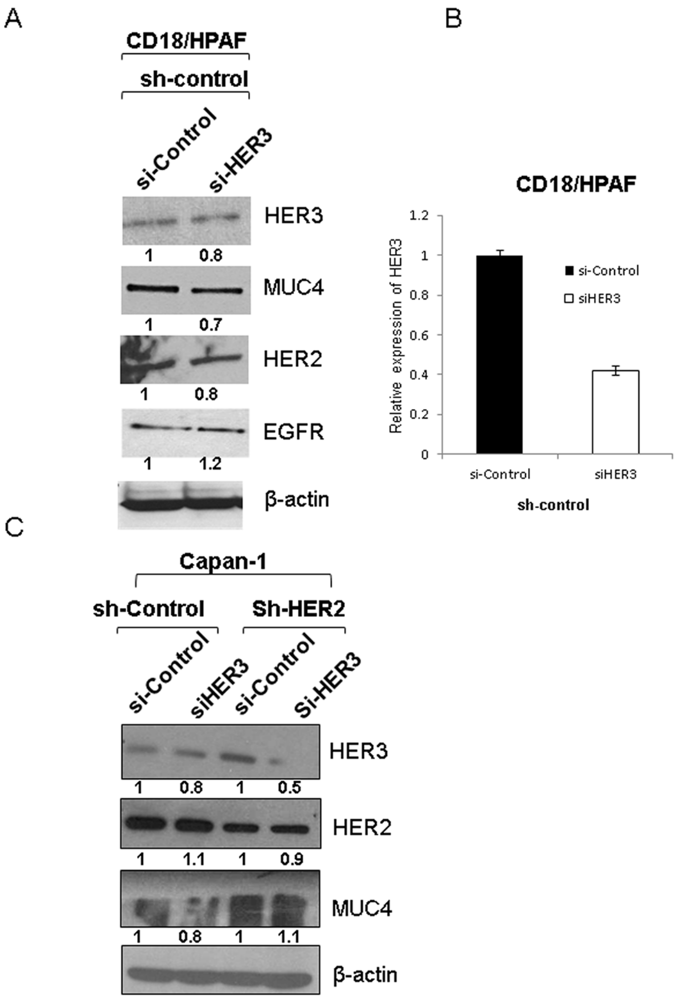

Supplementary Figure S4: Transient knockdown of HER3 in Capan-1 cells. The results show that the effect of HER3 knockdown on HER2 and MUC4 expression.
